# Dietary Nutrient Intake, Alcohol Metabolism, and Hangover Severity

**DOI:** 10.3390/jcm8091316

**Published:** 2019-08-27

**Authors:** Joris C. Verster, Sterre A. Vermeulen, Aurora J. A. E. van de Loo, Stephanie Balikji, Aletta D. Kraneveld, Johan Garssen, Andrew Scholey

**Affiliations:** 1 Division of Pharmacology, Utrecht Institute for Pharmaceutical Sciences, Utrecht University, 3584CG Utrecht, The Netherlands; 2 Institute for Risk Assessment Sciences (IRAS), Utrecht University, 3584CM Utrecht, The Netherlands; 3 Centre for Human Psychopharmacology, Swinburne University, Melbourne, VIC 3122, Australia; 4 Nutricia Research, 3584CT Utrecht, The Netherlands

**Keywords:** alcohol, hangover, nutrients, zinc, nicotinic acid, bootstrapping

## Abstract

Several dietary components have been shown to influence alcohol metabolism and thereby potentially affect the development of a hangover. From the literature, it is evident that dietary nicotinic acid and zinc play a pivotal role in the oxidation of ethanol into acetaldehyde. The aim of the current study was to associate dietary intake of nicotinic acid and zinc with hangover severity. To this end, data from *n* = 23 healthy social drinkers who participated in a naturalistic hangover study were analyzed. *n* = 10 of them reported to be hangover-resistant (the control group), whereas *n* = 13 reported to have regular hangovers (the hangover-sensitive group). Two 24 h dietary recall records were completed, one for the day of alcohol consumption and another one for an alcohol-free control day. Dietary nutrient intake was averaged and did not significantly differ between hangover-sensitive and hangover-resistant drinkers. For the hangover-sensitive drinkers, partial correlations with overall hangover severity were computed, controlling for estimated blood alcohol concentration. A bootstrapping technique was applied to account for the relatively small sample size. The results showed that dietary intake of nicotinic acid (r_PB_ = −0.521) and zinc (r_PB_ = −0.341) were significantly and negatively associated (*p* < 0.002) with overall hangover severity. Dietary zinc intake was also significantly and negatively associated with severity of vomiting (r_PB_ = −0.577, *p* < 0.002). No significant associations with hangover severity were found for other nutrients, such as fat and fibers. In conclusion, this study suggests that social drinkers who have a higher dietary intake of nicotinic acid and zinc report significantly less severe hangovers. As hangover-resistant and hangover-sensitive drinkers had a similar dietary nutrient intake, the claim of being hangover-resistant must be based on other unknown biopsychosocial factors. These findings should be replicated in a larger sample and include more elaborate food frequency questionnaires or nutrient-specific dietary intake records for zinc and nicotinic acid, and preferably accompanied by nutrient assessments in urine and/or blood.

## 1. Introduction

The alcohol hangover refers to the combination of mental and physical symptoms, experienced the day after a single episode of heavy drinking, starting when blood alcohol concentration (BAC) approaches zero [1]. The pathology underlying alcohol hangover is not well understood [2,3], and increasingly the subject of scientific investigation. In parallel, research has also been directed at the development of alcohol hangover treatments. This has led to the study of compounds that can influence the immune response to heavy alcohol consumption (which is assumed to contribute to alcohol hangover). Several hangover treatments have been reported to attenuate the rise in blood cytokines concentrations seen after heavy drinking, as well as reducing selective next day hangover symptoms. For example, Kim et al. [4] showed that *Hovenia dulcis Thunb* fruit extract (containing dihydromyricetin and heteropolysaccharides) significantly reduced blood cytokine concentrations that were increased due to heavy drinking. This was accompanied by a significant reduction in overall hangover severity. Interestingly, *Hovenia dulcis Thunb* fruit extract had no effect on alcohol metabolism (i.e., blood ethanol and acetaldehyde concentrations were not different from the alcohol only condition).

A different approach is to develop compounds that accelerate alcohol metabolism. The rationale for this approach is that more rapid elimination of ethanol and acetaldehyde could reduce the presence and severity of alcohol hangover symptoms. This hypothesis is supported by recent research showing that urine ethanol concentration was significantly lower in drinkers claiming to have no hangover after heavy alcohol consumption compared to drinkers who reported a hangover [5]. Although overall hangover severity was positively associated with the amount of ethanol found in urine of those who reported having a hangover, the partial correlation controlling for eBAC was not statistically significant. Nevertheless, this finding suggests that drinkers with slower alcohol metabolism, i.e., those with more ethanol in their urine, report significantly more frequent, and more severe hangovers. In other words, speeding up alcohol metabolism may have a beneficial effect on reducing hangover severity.

Another approach to the development of hangover treatments is to examine whether dietary nutrient intake has an effect on hangover severity. Two review papers have addressed this [6,7]. Firstly, Min et al. [6] argued that various minerals, including selenium, zinc, copper, vanadium, iron, and magnesium, may have a direct effect on either alcohol metabolism, on glutamatergic activity, or may influence the presence and severity of alcohol hangover via their antioxidant and/or anti-inflammatory properties. Secondly, Wang et al. [7] described the proposed mechanism of action of a number of natural products that might alleviate alcohol hangover symptoms. Both authors stressed that their hypotheses were based on limited animal research, and that research in humans is necessary to investigate the actual efficacy of minerals and herbal supplements in reducing or preventing alcohol hangover symptoms.

The scientific literature indicates that food intake can indeed have a significant effect on alcohol metabolism, both quantitatively and qualitatively. For example, relative to fasting, the consumption of foods before or together with alcohol reduces peak blood alcohol concentration (BAC), decreases absorption and slows metabolism [8,9,10]. In particular, ‘high-energy’ meals may slow down alcohol metabolism and reduce subjective intoxication [11,12,13]. Specific food products or nutrients have also been investigated. However, mixed results have been reported in relation to alcohol metabolism. For example, Kim et al. [14] found that consuming a mixed fruit and vegetable juice (Angelica keiskei/green grape/pear juice) significantly reduced peak BAC. In another study, Hong [15] examined the effect of a purported hangover treatment (DTS20, a mixture that consists of *Viscum album L.*, *Lycium chinense L.*, *Inonotus obliquus*, and *Acanthopanax senticosus H.*). The proposed active ingredients of DTS20 are sugar, uronic acid, and polyphenols. Relative to placebo, DTS20 significantly reduced BAC at 2 h after drinking alcohol in the form of Soju. The reduction in blood acetaldehyde levels, however, did not reach statistical significance. Taken together, there is limited evidence to date to support the notion that acute intake of specific nutrients can alter alcohol metabolism. Thus, further research into nutrients that can accelerate alcohol metabolism is warranted. Research on the possible impact of regular nutrient intake on hangover susceptibility to hangovers or relating nutrient intake to hangover symptom severity is currently lacking.

**Figure 1:**
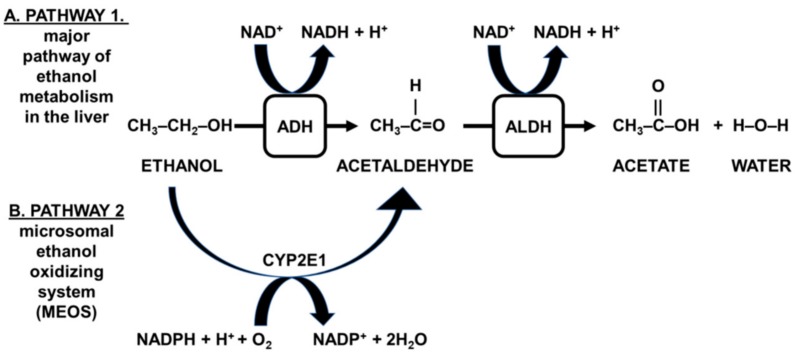
Pathways involved in alcohol metabolism. In the major metabolic pathway (**A**) ethanol is oxidized into acetaldehyde. This oxidative process is facilitated by alcohol dehydrogenase (ADH), which is present in high concentration in the cytosol of hepatocytes. In this second step, acetaldehyde enters the mitochondria where it is oxidized into acetate and water. This process is facilitated by mitochondrial aldehyde dehydrogenase (ALDH). For both steps, nicotinamide adenine dinucleotide (NAD^+^) is essential to provide the necessary energy for the conversion, which becomes available when NAD^+^ is converted into NADH + H^+^. A second major pathway for alcohol breakdown, especially active in subjects who chronically drink alcohol, is the microsomal ethanol oxidizing system (MEOS, see (**B**)). The reaction is catalyzed by CYP2E1 and requires nicotinamide adenine dinucleotide phosphate (NADP^+^) instead of NAD^+^ to convert ethanol into acetaldehyde.

Alcohol is metabolized primarily in the liver via a two-step reaction (see Figure 1) [16,17]. First, ethanol is oxidized into acetaldehyde, which is highly toxic. Although the first step in alcohol metabolism is reversible, acetaldehyde is usually metabolized rapidly. In this second step, acetaldehyde enters the mitochondria where it is oxidized into acetate and water. This process is facilitated by mitochondrial aldehyde dehydrogenase (ALDH). For both steps, nicotinamide adenine dinucleotide (NAD^+^) is essential to provide the necessary energy for the conversion, which becomes available when NAD^+^ is converted into NADH + H^+^.

NADP^+^ can be formed from NAD^+^, and differs from NAD^+^ in the presence of an additional phosphate group [18]. The conversion of acetaldehyde into acetate and water is similar to that overserved in the major alcohol metabolism pathway and requires NAD^+^. A third minor pathway oxidizes ethanol into acetaldehyde via catalase (not shown in Figure 1) [17]. Together, the oxidative pathways account for over 90% of alcohol elimination [19]. Thus, ADH thus plays a vital role in alcohol metabolism. 

Two nutrients are known to play an important role in alcohol metabolism, namely nicotinic acid and zinc [20,21]. Dietary intake of these micronutrients is necessary, as the body is unable to synthesize them itself [22,23]. Other nutrients do not seem to have an important direct influence on alcohol metabolism. 

Zinc (Zn^2+^) is absorbed from the small intestine. Most zinc can be found in tissue, with only 0.1% of total bodily zinc present in blood, where most (~70%) is bound to serum albumin [24]. From here zinc is transported as needed to body tissues. Since zinc is essential in the conversion of ethanol into acetaldehyde [20,21], we hypothesize that drinkers who consume abundant amounts of dietary zinc metabolize alcohol faster than those who consume relatively lower levels. 

Niacin and its equivalents are the main dietary source of NAD^+^. Tryptophan is also a source of NAD^+^, and its relative contribution is estimated as 60 mg of tryptophan equaling 1 mg of nicotinic acid and other niacin equivalents, although a 30% individual variability in the conversion from tryptophan into nicotinic acid has been observed [25]. For the MEOS alcohol metabolism pathway (see Figure 1), NADP^+^ is required. Nicotinic acid and its equivalents are the dietary sources of both NAD^+^ and NADP^+^, which together catalyze alcohol metabolism.

We hypothesize that when abundant amounts of nicotinic acid are present in the daily diet of a drinker, alcohol is metabolized faster than in drinkers who have lower levels of dietary nicotinic acid intake. To investigate the hypothesis that higher levels of dietary nicotinic acid and zinc may be protective against alcohol hangover of healthy social drinkers, dietary food intake was recorded during an experimental hangover study. Dietary nicotinic acid and zinc intake were computed and related to hangover severity.

## 2. Materials and Methods

A naturalistic study was conducted, including a ‘control day’ (no alcohol consumed) and a ‘hangover day’ (alcohol consumed the evening before), separated by approximately one week. Written informed consent was obtained from each participant, and The University of Groningen Psychology Ethics Committee approved the study.

### 2.1. Subjects

*n* = 23 subjects participated in the study. Of them, *n* = 13 subjects (*n* = 7 men and *n* = 6 women) regularly have hangovers when they consume alcohol and *n* = 10 (*n* = 4 men and *n* = 6 women) drinkers claimed to be hangover-resistant. They were recruited by local advertisement at Utrecht University. After indicating interest, participants were contacted over the phone and underwent an initial screening. Subjects were included if they were between 18–30 years old, mentally and physically healthy, social drinkers. Recreational drug users and smokers were excluded from participation. Further exclusion criteria comprised a positive urine drug or pregnancy screen, the use of medicinal drugs (including over-the-counter pain killers), and alcohol consumption within 24 h before the start of the control test day. To maximize the likelihood that subjects would actually have a hangover during the study, subjects were included only if it was demonstrated that they consume sufficient amounts of alcohol that can produce a hangover per se. To check this, estimated blood alcohol concentration (eBAC) was calculated for the alcohol consumption they reported for ‘a regular night out’. This was done by applying a modified Widmark equation [26], taking into account gender and body weight. To be enrolled in the study, an eBAC of 0.08% or higher was required, based on hangover susceptibility likelihood calculations by Verster et al. [27] and Kruisselbrink et al. [28].

### 2.2. Procedures

The naturalistic design was chosen to closely mimic a realistic real-life hangover experience which has relatively higher ecological validity compared with other methodologies [29]. Researchers were not present during alcohol consumption, and thus had no influence on the participants’ (drinking) behavior. Participants, therefore, dictated their own time period of drinking, types of alcoholic beverages consumed, and their activities during drinking (e.g., staying at home, going to a bar, dancing, etc.). Subjects were asked not to change their lifestyle while participating in the study. They were asked to refrain from consuming any alcohol three days prior to the control day. Both test days started at 9 a.m. Subjects completed a 24 h dietary recall diary [30] and the presence and severity of hangover symptoms were assessed. A urine drug screen (Instant-View, determining the presence of amphetamines, barbiturates, cannabinoids, benzodiazepines, cocaine, and opiates) was conducted according to the manufacturer’s instructions (Alfa Scientific Designs, Inc., Poway, CA, USA). 

### 2.3. Alcohol Consumption and Hangover Severity

At 09:45 a.m., previous night alcohol consumption was recorded (number of units), including the times at which drinking commenced and ceased. The adjusted Widmark formula [26] was used to calculate eBAC. Overall hangover severity was assessed with a single one-item rating on an 11-point scale ranging from 0 (absent) to 10 (extreme) [31]. In addition, using the same scale, severity of 23 hangover symptoms was assessed, including headache, nausea, concentration problems, regret, sleepiness, heart pounding, vomiting, being tired, shaking/shivering, clumsiness, weakness, dizziness, apathy, sweating, stomach pain, confusion, sensitivity to light, sensitivity to sound, thirst, heart racing, anxiety, depression, and reduced appetite. 

### 2.4. Dietary Recall

The participants were asked to complete a 24-h dietary recall diary [31]. This assessed the 24 h dietary intake before both test days. Participants wrote down the amount and type of food as precisely as possible. Subjects were asked to record all food and beverages consumed for breakfast, lunch, dinner, and ‘in-between’ during the past 24 h. They were instructed to write down the time of consumption and the amount consumed (i.e., an estimate of the portion size). Examples were provided on how to complete the diary. Participants were urged not to forget smaller, ‘less significant’ food items, such as pieces of candy and to include details such as whether or not they buttered any bread consumed. Nutrient calculations were performed using the ‘eetmeter’ (‘eating meter’ in English) [32], developed by the ’Voedingscentrum’ of ‘Rijksinstituut voor Volksgezondheid en Milieu’ (RIVM). The assessments take into account quantities, frequency, and portion sizes of consumed food and beverages. Nutrients obtained from alcoholic and non-alcoholic beverages, consumed during the drinking session, were included in the calculation models. The ‘eetmeter’ provided data on nutrient intake of nicotinic acid, zinc, total fat (triglycerides, esters derived from glycerol and fatty acids, and fatty constituents such as phosphatides and sterols), saturated fat (total of saturated fatty acids), carbohydrates (total of mono- and disaccharides, starch, dextrin, and glycogen), proteins, fibers, water, sugar, vitamin A (retinol), vitamin B1 (thiamine), vitamin B2 (riboflavin), vitamin B6 (pyridoxine, including pyridoxal and pyridoxamine), vitamin B11 (folic acid), vitamin B12 (cobalamins), vitamin C (ascorbic acid, including L-ascorbic acid and L-dehydro-ascorbic acid), vitamin D (cholecalciferol and 25-hydroxy vitamin D), vitamin E, salt, sodium, potassium, calcium, magnesium, iron, selenium, iodine, and phosphorus [33]. Dietary intake was computed for the alcohol and the control day. The average dietary intake of the two days was computed to better represent daily dietary nutrient intake.

### 2.5. Statistical Analysis

Statistical analyses were conducted with SPSS, version 25 (Armonk, IBM Corp, New York, NY, USA). Mean and standard deviation (SD) were computed for each variable. Hangover-sensitive drinkers were included in the analysis if they had an overall hangover score of 2 or higher. Hangover-resistant drinkers were included in the analysis is they had an overall hangover score of 0 or 1. Firstly, nutrient intake was computed separately for the alcohol test day and alcohol-free control day respectively. These were combined into a two-day average score to better reflect regular nutrient intake. Secondly, dietary nutrient intake of hangover-sensitive and hangover-resistant drinkers was compared. Third, dietary nutrient intake of hangover-sensitive drinkers was correlated with overall hangover severity, by computing partial correlations (r_P_), controlling for eBAC. This approach controlled for the different alcohol consumption levels between participants. Thirdly, nutrient intake levels that correlated significantly with overall hangover severity were further correlated with the 23 individual hangover severity measures, again by computing r_P_, controlling for eBAC.

**Figure 2:**
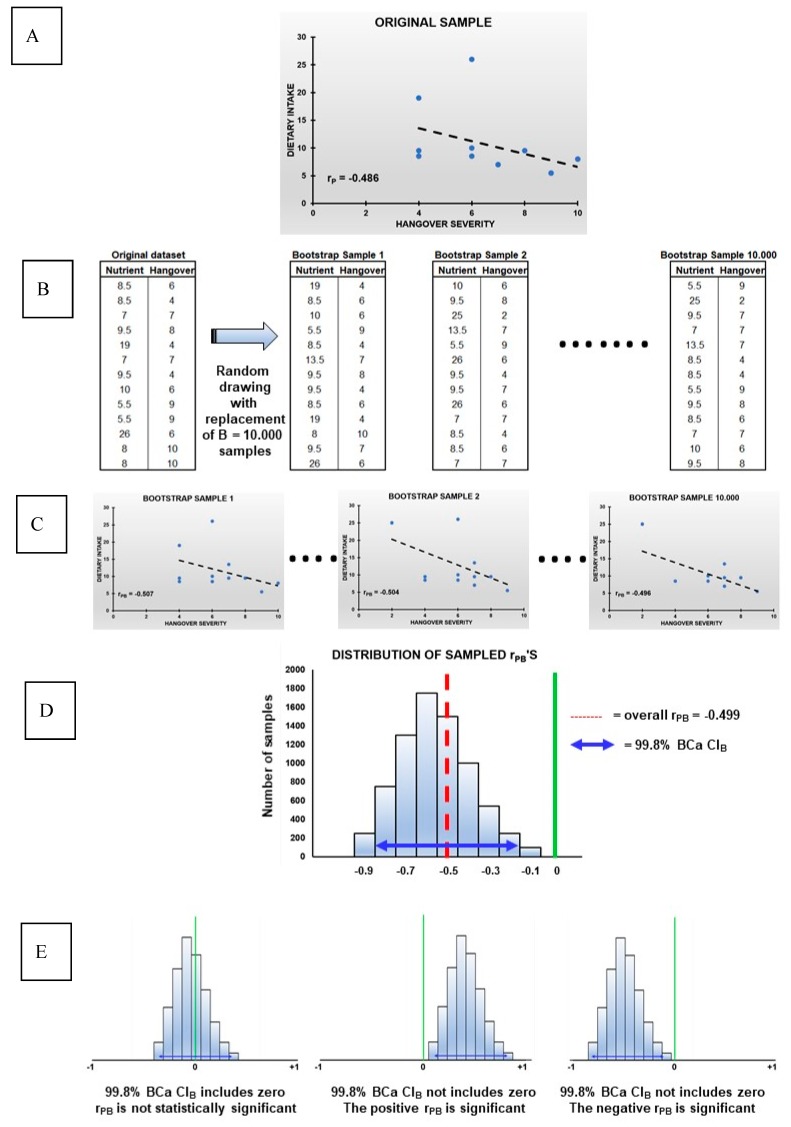
The bootstrapping technique. (**A**) original sample, (**B**) new datasets, (**C**) B = 10.000 samples, (**D**) Composing a histogram of all rPB, (**E**) rPB is considered statistically significant. Abbreviations: r_P_ = partial correlation, r_PB_ = bootstrapped partial correlation, BCa = bias-corrected and accelerated, C_IB_ = bootstrapped confidence interval.

To further examine the data and account for the relatively small sample size, a bootstrapping analysis [34,35] was conducted to simulate the population distributions of the partial correlations (r_P_). Bootstrapping and interpretation of its results are summarized in Figure 2. In bootstrapping, data from the original sample (Figure 2A) are used to generate B new datasets (Figure 2B). The new samples have the same sample size as the original sample. The new samples are constructed by randomly drawing cases (resampling), with replacement, from the original sample. In the current analysis, B = 10.000 samples (of *n* = 13 subjects each) were created (Figure 2C), a recommended resampling size for bootstrapped CI estimation [36]. For each of the bootstrap samples a new r_P_ is then computed (the bootstrapped partial correlation, denoted as r_PB_). Composing a histogram of all r_PB_ (Figure 2D) results in a histogram with a normal distribution that usually mimics the population distribution [36]. Subsequently, it can then be calculated how much the r_PB_’s vary across the bootstrap samples (Standard Error, SE). The reported ‘Bias’ measure represents the deviation of the overall r_PB_ from the r_P_ that was obtained from the original sample [37]. To compute the corresponding bootstrapped confidence interval (CIB), a Bias Corrected and accelerated (BCa) correction was applied [38]. This correction was applied to adjust for the observed Bias, and to account for potential skewness of the bootstrap distribution (operationalized as SE). In the case of (partial) correlations, CIB’s can range from −1 to +1. Narrow CIB’s imply greater precision, and if the CIB does not include zero, the r_PB_ is considered statistically significant (Figure 2E). Usually, 95% CIB’s are computed (corresponding to a significance level of α = 5%). However, to correct for the multiple comparisons in the current study, a 99.8% CIB was computed (corresponding to a significance level of *p* < 0.002). 

## 3. Results

Data from *n* = 13 hangover-sensitive subjects (seven men and six women) were included in the analysis. Their mean (±SD) age was 20.8 (±1.4) years old. Their mean height and weight were 1.76 (±0.07) m and 70.8 (±7.9) kg, respectively. They consumed a mean of 11.3 (±3.8) alcoholic drinks on the alcohol test day, resulting in an eBAC of 0.20 (±0.07)%, and a next-day overall hangover score of 6.2 (±2.2). *n* = 10 hangover-resistant drinkers (four men and six women) served as control group in this study. Demographics and drinking variable outcomes were comparable to those of the hangover-sensitive group. Their mean (±SD) age was 21.2 (±2.4) years old. Their mean height and weight were 1.76 (±0.11) m and 73.0 (±15.9) kg, respectively. They consumed a mean of 13.0 (±6.2) alcoholic drinks on the alcohol test day, resulting in an eBAC of 0.22 (±0.12)%, and a next-day overall hangover score of 0.2 (±0.4). Dietary nutrient intake of hangover-sensitive drinkers did not significantly differ from that of hangover-resistant drinkers. Moreover, alcohol consumption and eBAC did not significantly differ between the two groups. The next statistical analysis was, therefore, conducted only for the hangover-sensitive group. Dietary nutrient intake on the alcohol and control day are summarized in Table 1.

On the alcohol day, alcohol was consumed, and this was accompanied by significantly increased water intake (included in the beverages). As a result, the energy intake on the alcohol day was also significantly greater compared to the control day. However, dietary nutrient intake data show no significant differences between the alcohol day and the control day. Therefore, the statistical analysis that follows the two-day average nutrient intake is used. The association of dietary nutrient intake and overall hangover severity is summarized in Table 2.

**Figure 3:**
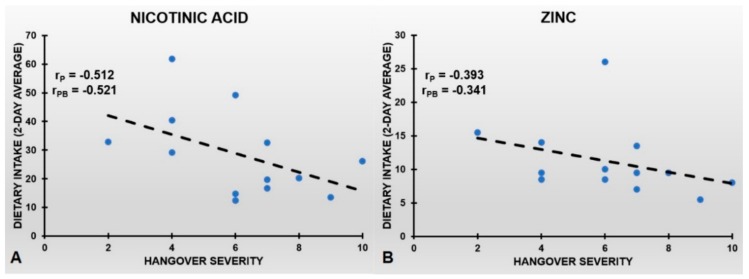
The negative association of dietary nicotinic acid intake (two-day average) and dietary zinc intake with overall hangover severity. Figure 3 shows the partial correlation (r_P_), controlled for eBAC, between (**A**) dietary nicotinic acid and (**B**) zinc intake (two-day average) and overall hangover severity.

The association between both the two-day average dietary nicotinic acid and zinc intake and hangover severity is shown in Figure 3. It is evident from Figure 3 that higher levels of dietary nicotinic acid and zinc are associated with less severe alcohol hangovers. After bootstrapping with 10.000 samples the r_PB_’s were statistically significant (*p* < 0.002). Increasing the bootstrap sample size to B = 100.000 samples did not alter the results. Bootstrapping analysis of other nutrients revealed no significant r_PB_s between overall hangover severity and dietary nutrient intake (see Table 2).

**Figure 4:**
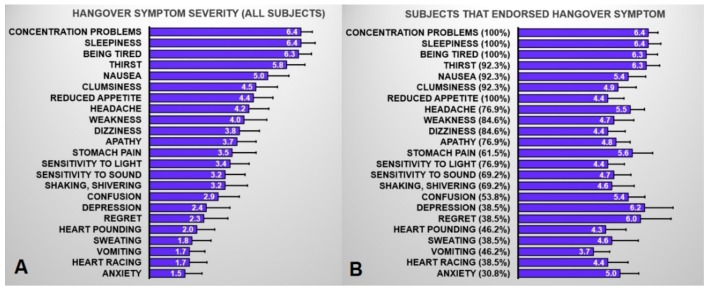
Hangover symptom severity. The average score across all subjects is shown for each hangover symptom in (**A**). (**B**) shows the hangover severity reported by only those subjects that endorsed the hangover symptom. Error bars represent the standard error.

Figure 4 summarizes the mean (SD) severity scores on the individual hangover symptoms. A bootstrapping analysis was conducted to investigate the r_PB_’s, controlling for eBAC, between individual hangover symptom severity scores and dietary intake of nicotinic acid and zinc. The bootstrapping results are summarized in Table 3 and Table 4.

The r_PB_’s between hangover symptom severity and dietary intake of nicotinic acid were negative, suggesting that increased dietary nicotinic acid intake is beneficial for reducing hangover symptom severity. However, in contrast to overall hangover severity, the r_PB_’s with individual hangover symptoms were not statistically significant (see Table 3).

**Figure 5:**
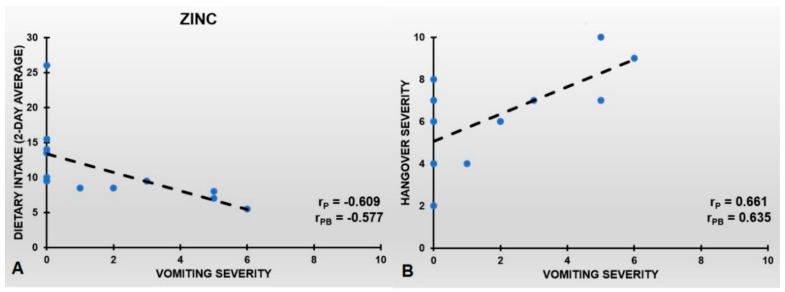
Severity of vomiting, dietary zinc intake, and overall hangover severity. (**A**) shows the partial correlation (r_P_), controlled for eBAC, between dietary zinc intake (two-day average) and vomiting severity during alcohol hangover. (**B**) shows the r_P_, controlled for eBAC, between overall hangover severity and vomiting severity. After bootstrapping, both the corresponding r_PB_’s are significant at the *p* < 0.002 level.

The r_PB_’s between hangover symptom severity and dietary intake of zinc were also negative, suggesting that also increased dietary nicotinic acid intake might be beneficial for reducing hangover symptom severity. Dietary zinc intake (two-day average) was significantly associated with the severity of vomiting (see Table 4 and Figure 5A). The negative r_PB_ implies that higher levels of dietary zinc are associated with less severe vomiting. Increasing the bootstrap sample size to B = 100.000 samples did not alter the results. Figure 5B shows that the severity of vomiting is an important determinant of overall hangover severity (r_P_ = 0.661, *p* = 0.019, r_PB_ = 0.635, significant at the *p* < 0.002 level). No significant r_PB_’s were found between two-day average dietary zinc intake and the severity of the other 21 hangover symptoms (see Table 4).

Finally, although the number of men (*n* = 7) and women (*n* = 6) did not allow for reliable sex comparisons, we conducted some exploratory analysis. The analysis revealed that men had a higher dietary nicotinic acid intake than women (38.0 mg and 17.2 mg, respectively, *p* = 0.002) as well as a higher intake of zinc (13.8 mg and 8.1 mg, respectively, *p* = 0.022). The differences did not reach significance at the *p* < 0.0017 level. Men reported less severe hangovers than women (scores of 5.0 and 7.5, respectively, *p* = 0.073), while their eBAC did not differ significantly (eBAC 0.20% and 0.19%, respectively, *p* = 0.628). The partial correlations between dietary nicotinic acid and zinc intake and hangover severity calculated for men and women separately were not statistically significant. 

## 4. Discussion

The current study demonstrated that dietary nicotinic acid and zinc intake were significantly and negatively associated with overall hangover severity. Both nutrients are essential in effective alcohol metabolism and, therefore, the current findings suggest that more rapid and efficient oxidation of ethanol into acetaldehyde, and acetaldehyde into acetate, may be associated with less severe hangovers. There was no similar association found for the other nutrients that were examined. 

Sufficient dietary intake of zinc and nicotinic acid are important to maintain health. Examples of food rich in zinc are meat, shellfish (e.g., oysters), and legumes, such as lentils and beans. The recommended dietary allowance for zinc is 11 mg per day for men and 8 mg per day for women [39]. Deficiency of zinc can have serious health consequences and negatively impact immune defense [23]. Zinc deficiency is relatively more common among the elderly. For example, a US study found that only 42.5% of the elderly had an adequate level of dietary zinc intake [40]. 

Examples of food rich in nicotinic acid include those containing high levels of niacin or tryptophan such as meat, fish and poultry, avocado, peanuts, whole grains, and mushrooms. The recommended dietary allowance (RDA) for niacin and its equivalents is 16 mg per day for men and 14 mg per day for women [41,42]. Pellagra (pigmented skin rash) is a common consequence of severe niacin deficiency [43], and, although uncommon in the general population of Western countries, it is seen among chronic alcoholics [41]. In this context, niacin supplementation has been suggested as a treatment for alcoholism [44]. Our data suggest that this should be explored in more detail in different types of social drinkers, given the obvious relationship between frequent heavy drinking and hangover frequency.

The current finding may have implications for the development of effective hangover treatments. Although there is a buoyant market for so-called hangover treatment among social drinkers [45], currently they lack robust evidence of efficacy [46,47,48]. Several newly developed putative hangover treatments are comprised of natural ingredients, such as plant extracts, herbs, minerals and vitamins [48]. For example, studies were conducted to investigate the effects of, Korean pear juice [49] and red ginseng [50], which both showed some positive effects in reducing hangover severity. However, other products, such as artichoke extract [51], showed no beneficial effects on hangover. Kelly et al. [52] found that intravenous vitamin B complex and Vitamin C had no significant effect on alcohol metabolism. Kahn et al. [53] reported that pyritinol (1200 mg oral vitamin B6) significantly reduced the number of reported hangover symptoms, but unfortunately, no assessments were conducted with regard to the severity of hangover symptoms. Laas [54] conducted a double-blind placebo-controlled study to examine the efficacy of ‘Morning Fit’ (dried yeast, thiamine nitrate (Vitamin B1), pyridozine hydrochloride (Vitamin B6) and riboflavin (Vitamin B2) and found no significant differences in either blood alcohol or acetaldehyde concentrations between the Morning Fit and placebo condition. Although significant improvements were reported for certain individual symptoms, namely ‘uncomfortable feelings’, ‘restlessness’, and ‘impatience’, no significant improvement was found on general wellbeing. A study conducted by Ylikahri et al. found no significant effect of sugars such as fructose and glucose on alcohol metabolism or hangover severity [55]. A more recent study by Bang et al. examined the effects on hangover of a polysaccharide-rich extract of *Acanthopanax senticosus* (PEA) [56]. While blood sample analysis revealed no significant effect on alcohol metabolism, PEA did, however, significantly reduce alcohol-induced next-day changes in glucose and c-reactive protein levels (i.e., it was effective in reducing alcohol-induced hypoglycemia and inhibiting the inflammatory response, respectively). Overall hangover severity, and the individual hangover symptoms, such as tiredness, headache, dizziness, stomachache and nausea, significantly improved after administering PEA.

Taken together, there is mixed evidence on acute effects of dietary nutrients on the presence and severity of hangover symptoms. The current findings are also in contrast to anecdotal evidence that suggests that taking fiber-rich food, consuming water, or eating fat-rich meals may reduce the severity of alcohol hangovers.

In the current study, dietary zinc and nicotinic acid intake (or any other nutrient that was assessed) did not significantly differ between hangover-sensitive drinkers and hangover-resistant drinkers. Thus, it is unlikely that supplementing diet with high levels of nicotinic acid and zinc makes a hangover sensitive drinker immune to hangovers. However, the data of hangover-sensitive drinkers clearly show that higher dietary intake of both nutrients is associated with experiencing less severe hangovers. The issue of drinkers claiming hangover resistance is a complex one. Data show that this claim heavily depends on how much alcohol drinkers consume, but even at higher eBAC levels a small proportion of drinkers claim not to have hangovers [27,28]. At the same time, other hangover research showed no significant differences between the two groups of drinkers on several biomarkers such as urine ethyl glucuronide (EtG) and ethyl sulfate (EtS) [57] or methanol [58], saliva cytokine levels [59], sensitivity to acute alcohol effects [60], demographics [31], or psychological characteristics such as mental resilience [61]. Additionally, the current study could not differentiate hangover-sensitive and hangover-resistant drinkers based on their dietary nutrient intake. Thus, there must be different unknown biopsychosocial factors (e.g., alexithymia) that may explain why some drinkers claim to be hangover-resistant. Research did show that experiencing alcohol hangovers (compared to claiming to be resistant) was associated with significantly poorer self-reported immune function [62] and having higher urine ethanol concentrations during the hangover state [5]. Future research should further investigate the puzzling phenomenon of hangover-resistance. 

The study has several limitations. Firstly, it had a small sample size. Future research should, therefore, aim to replicate the current findings in larger samples. The use of bootstrapping techniques in hypothesis testing is increasingly popular [36] and was used in the current analysis to mitigate the small sample size. Secondly, the sample size was too small to reliably assess possible gender differences. Explorative analysis revealed that men had a higher intake of dietary nicotinic acid and zinc. Moreover, women reported non-significantly higher hangover severity than men, and eBAC levels did not differ significantly. These findings are in line with a recent analysis showing that the presence and severity of hangover symptoms experienced at the same eBAC levels show no relevant sex differences [63]. However, studies have shown sex differences in cognitive functioning and driving performance the morning following bedtime administration of other psychoactive drugs, such as hypnotics [64]. Therefore, future replication research with larger sample size is required to further investigate possible sex differences during the hangover state in these domains. Thirdly, eBAC assessments were based on subjective retrospective recall of the number of alcoholic drinks consumed. These reports may to some extent be inaccurate. In naturalistic study designs, researchers are not present during the drinking occasion, to ensure the real-life ‘natural’ drinking setting, and retrospective assessments of alcohol consumption are common practice. However, very recently real-time objective BAC assessment devices have been developed that are capable of continuously recording transdermal BAC. It would be useful to use such devices in future naturalistic studies. Fourthly, there were many statistical comparisons made in the analysis. Although the primary aim was to investigate the association between dietary nicotinic acid and zinc intake with overall hangover severity, we also collected data on 25 other nutrients and 23 individual hangover symptoms. A strict Bonferroni’s correction (*p* < 0.002) was applied to account for this, and therefore we are confident with the reported statistical significance thresholds.

Dietary nutrient intake was collected via 24-h dietary recall records. These were completed by the subjects the day after drinking, and thus recall bias may have resulted in underestimation or overestimation of food portions, omitting food items or erroneously adding others. On the other hand, there was good correspondence between the diary measures across the two collection periods. Furthermore, the group average nutrient intake in the current study corresponds well to large scale studies that assessed nutrient intake via elaborate food frequency questionnaires [41,42]. This provides some confidence that recall bias played a minor role in the current study. Clearly, future research should utilize more elaborate food frequency questionnaires, or nutrient-specific dietary records for nicotinic acid or zinc. Additionally, assessments of nutrient status in blood or urine would provide an objective measure of nutrient status. 

It is relevant to note that dietary nutrients can impact alcohol metabolism via the gut and oral microbiome. Dietary nutrient intake, as well as alcohol consumption, have an influence on the composition of the microbiome. Several studies have reported the effects of alcohol consumption and dietary intake on microbiota composition [65,66]. The effect of these on hangover is not well understood, but a high abundance of several microbiota, including *Rothia*, *Neisseria*, and *Streptococcus,* is associated with accelerated alcohol metabolism by producing relatively higher amounts of acetaldehyde [67]. Future research should investigate the relationship between the microbiome, and the presence and severity of alcohol hangover. Moreover, there are several other factors that may influence alcohol metabolism that were not assessed in the current study. These include, for example, various genetic and environmental factors, sex, age, race, biological rhythms (time of day), and medicinal and recreational drug use (e.g., compounds which inhibit ADH such as pyrazoles or isobutyramine), Antabuse (disulfiram, which inhibits the elimination of acetaldehyde), or other alcohols that compete with ethanol for ADH (e.g., methanol) [17]. These are also important topics for future research. 

Finally, the oxidative pathways account for over 90% of alcohol elimination [19]. In addition, there are also nonoxidative pathways for alcohol metabolism, producing metabolites such as ethyl-glucuronide (EtG), ethyl-sulfate (EtS), phosphatidyl-ethanol (PEth) and fatty acid ethyl ester (FAEE) [19]. As these pathways usually only process a very limited amount of alcohol, and thus the overall impact of nutrients on alcohol metabolism via these pathways can be considered as marginal, they were not taken into account in the current paper. 

## 5. Conclusions

In conclusion, this study suggests that social drinkers who have a higher dietary intake of nicotinic acid and zinc report significantly less severe hangovers. 

## Figures and Tables

**Table 1:** Dietary nutrient intake.

Nutrient	Control Day	Alcohol Day	*p*-Value	2-Day Average
Nicotinic acid (mg)	22.0 (17.1)	34.8 (19.4)	0.075	28.4 (14.9)
Zinc (mg)	9.7 (3.4)	12.6 (8.3)	0.134	11.2 (5.3)
Total fat (g)	83.6 (19.2)	103.1 (47.6)	0.221	93.4 (26.7)
Saturated fat (g)	27.3 (10.2)	35.5 (20.2)	0.249	31.4 (11.6)
Carbohydrates (g)	208.0 (57.8)	330.3 (86.7)	0.002	269.2 (62.2)
Proteins (g)	91.6 (38.3)	108.2 (69.0)	0.650	99.9 (45.9)
Fibers (g)	22.7 (7.5)	26.2 (5.5)	0.158	24.5 (4.8)
Salt (g)	7.5 (3.0)	10.1 (5.6)	0.055	8.8 (3.8)
Alcohol (g)	0 (0)	128.2 (45.3)	0.001 *	64.1 (22.6)
Water (ml)	942.7 (347.6)	3165.2 (933.9)	0.001 *	2053.9 (533.8)
Sodium (mg)	2989.3 (1121.5)	3843.4 (1946.8)	0.016	3416.3 (1396.2)
Potassium (mg)	2987.1 (695.6)	4228.5 (1287.1)	0.003	3607.8 (901.2)
Calcium (mg)	749.4 (356.7)	863.2 (472.4)	0.552	806.3 (285.3)
Magnesium (mg)	301.6 (86.9)	454.4 (168.3)	0.003	378.0 (111.4)
Iron (mg)	10.2 (2.2)	11.7 (4.7)	0.311	11.0 (2.9)
Selenium (mg)	52.8 (30.4)	60.8 (34.5)	0.916	56.8 (26.4)
Iodine (mg)	188.8 (51.4)	187.1 (76.6)	0.600	187.3 (52.6)
Phosphorus (mg)	1464.3 (504.9)	1622.2 (1134.5)	0.701	1543.3 (693.4)
Vitamin A (mg)	665.6 (640.6)	769.3 (521.8)	0.345	717.5 (451.8)
Vitamin B1 (mg)	0.9 (0.2)	1.2 (0.9)	0.421	1.1 (0.5)
Vitamin B2 (mg)	1.3 (0.5)	1.8 (0.9)	0.133	1.5 (0.5)
Vitamin B6 (mg)	1.7 (0.9)	2.7 (1.3)	0.011	2.2 (0.9)
Vitamin B11 (mg)	238.4 (100.8)	349.4 (129.0)	0.033	293.9 (86.3)
Vitamin B12 (mg)	7.4 (12.4)	4.3 (2.8)	0.861	5.8 (6.0)
Vitamin C (mg)	89.2 (52.6)	97.1 (95.4)	0.969	93.2 (65.4)
Vitamin D (mg)	2.0 (1.9)	3.1 (1.8)	0.084	2.5 (1.4)
Vitamin E (mg)	13.3 (5.7)	14.4 (7.0)	0.807	13.8 (4.7)
Energy (Kcal)	2003.7 (406.8)	3655.8 (1030.0)	0.001 *	2829.8 (621.1)

Related-samples Wilcoxon singed rank test. Results are significant if *p* < 0.0017, after Bonferroni’s correction for multiple comparisons, indicated by *.

**Table 2:** Association between dietary nutrient intake (two-day average) and overall hangover severity.

	Original Sample		Bootstrapping Results				
Nutrients	r_P_	*p*-Value	Bias	SE	r_PB_	Lower CI_B_ Limit	Upper CI_B_ Limit
Nicotinic acid *	−0.512	0.089	0.009	0.185	−0.521	−0.893	−0.032
Zinc *	−0.393	0.206	−0.052	0.219	−0.341	−0.829	−0.109
Total fat	−0.014	0.967	0.018	0.338	−0.032	−0.993	+0.998
Saturated fat	0.021	0.948	−0.011	0.331	−0.010	−0.917	+0.910
Carbohydrates	−0.223	0.485	−0.029	0.274	−0.204	−0.991	+0.940
Proteins	−0.285	0.370	0.000	0.269	−0.285	−0.997	+0.939
Fibers	−0.157	0.627	0.004	0.319	−0.161	−1.000	+0.999
Salt	−0.059	0.855	−0.073	0.424	−0.014	−0.981	+0.973
Alcohol	0.138	0.669	−0.026	0.294	0.112	−0.858	+0.910
Water	−0.103	0.749	−0.037	0.277	−0.066	−0.880	+0.764
Sodium	−0.157	0.626	−0.041	0.365	−0.116	−0.973	+0.856
Potassium	−0.409	0.187	0.004	0.266	−0.413	−0.985	+0.846
Calcium	−0.045	0.890	0.006	0.282	−0.051	−0.933	+0.946
Magnesium	−0.499	0.098	0.007	0.233	−0.506	−0.954	+0.328
Iron	−0.250	0.433	0.008	0.282	−0.258	−1.000	+1.000
Selenium	−0.356	0.256	0.017	0.292	−0.373	−0.971	+0.912
Iodine	−0.347	0.270	0.024	0.255	−0.371	−0.967	+0.885
Phosphorus	−0.355	0.258	0.034	0.247	−0.389	−1.000	+0.872
Vitamin A	0.125	0.698	0.012	0.243	0.137	−0.848	+0.962
Vitamin B1	0.019	0.952	0.017	0.272	0.036	−0.883	+0.874
Vitamin B2	−0.193	0.549	0.000	0.288	−0.193	−0.986	+0.723
Vitamin B6	−0.407	0.189	0.000	0.293	−0.407	−0.994	+0.950
Vitamin B11	−0.175	0.586	−0.014	0.354	−0.161	−0.996	+0.897
Vitamin B12	0.096	0.768	−0.046	0.279	0.050	−0.875	+0.697
Vitamin C	0.489	0.107	−0.125	0.417	0.364	−0.885	+0.947
Vitamin D	−0.146	0.651	−0.023	0.341	−0.123	−0.976	+0.956
Vitamin E	0.083	0.797	−0.003	0.273	0.080	−0.770	+0.917
Energy (Kcal)	−0.145	0.652	−0.009	0.259	−0.136	−0.961	+0.925

Bootstrapping was conducted with B = 10.000 samples. A BCa 99.8% CI_B_ (corresponding to *p* < 0.002) was used to correct for multiple comparisons. Partial correlations control for eBAC and are significant if the BCa 99.8% CI_B_ does not contain zero, indicated by *. Abbreviations: r_P_ = partial correlation, r_PB_ = bootstrapped partial correlation, eBAC = estimated blood alcohol concentration, SE = standard error, BCa = bias-corrected and accelerated, CI_B_ = bootstrapped confidence interval.

**Table 3:** Association between dietary nicotinic acid intake (two-day average) and hangover symptom severity.

	Original Sample		Bootstrapping Results				
Hangover Symptoms	r_P_	*p*-Value	Bias	SE	r_PB_	Lower CI_B_ Limit	Upper CI_B_ Limit
Concentration problems	−0.163	0.612	0.049	0.329	−0.212	−0.940	+0.948
Sleepiness	−0.248	0.437	−0.022	0.386	−0.226	−0.985	+0.885
Being tired	−0.448	0.145	0.043	0.348	−0.531	−1.000	+0.998
Thirst	−0.157	0.625	−0.013	0.255	−0.144	−0.897	+0.634
Nausea	−0.447	0.145	0.103	0.347	−0.550	−1.000	+1.000
Clumsiness	−0.272	0.392	0.029	0.302	−0.301	−0.975	+0.942
Reduced appetite	−0.181	0.573	0.080	0.360	−0.261	−1.000	+0.998
Headache	−0.561	0.058	0.049	0.234	−0.610	−0.987	+0.911
Weakness	−0.141	0.662	0.028	0.348	−0.169	−0.998	+1.000
Dizziness	−0.281	0.376	0.062	0.334	−0.343	−0.940	+0.792
Apathy	−0.303	0.339	0.120	0.419	−0.423	−0.987	+0.994
Stomach pain	−0.363	0.246	0.027	0.299	−0.390	−0.973	+0.813
Sensitivity to light	−0.352	0.262	0.082	0.345	−0.434	−0.985	+0.919
Sensitivity to sound	−0.406	0.190	0.064	0.349	−0.470	−1.000	+0.865
Shaking, shivering	−0.430	0.163	0.018	0.248	−0.448	−0.999	+0.940
Confusion	−0.204	0.524	0.047	0.334	−0.251	−0.999	+0.987
Depression	−0.335	0.287	0.061	0.354	−0.396	−0.982	+0.961
Regret	−0.324	0.304	0.078	0.365	−0.402	−0.994	+0.998
Heart pounding	−0.514	0.087	0.054	0.282	−0.568	−1.000	+0.997
Sweating	−0.347	0.268	0.071	0.366	−0.418	−0.994	+0.973
Vomiting	−0.506	0.093	0.016	0.235	−0.522	−0.999	+0.985
Heart racing	−0.439	0.154	0.066	0.325	−0.505	−1.000	+0.980
Anxiety	−0.379	0.224	0.059	0.346	−0.438	−0.977	+0.842

Reported r and *p*-value are from the original r_P_, controlling for eBAC. Bootstrapping was conducted with B = 10.000 samples. A 99.8% CI_B_ (corresponding to *p* < 0.002) was used to correct for multiple comparisons. None of the correlations are significant, as their BCa 99.8% CI_B_ contains zero. Abbreviations: r_P_ = partial correlation, eBAC = estimated blood alcohol concentration, SE = standard error, BCa = bias-corrected and accelerated, CI_B_ = bootstrapped confidence interval.

**Table 4:** Association between dietary zinc intake (two-day average) and hangover symptom severity.

	Original Sample		Bootstrapping Results				
Hangover Symptoms	r_P_	*p*-Value	Bias	SE	r_PB_	Lower CI_B_ Limit	Upper CI_B_ Limit
Concentration problems	−0.128	0.691	−0.007	0.314	−0.121	−0.854	+0.887
Sleepiness	−0.055	0.865	−0.072	0.326	0.023	−0.855	+0.741
Being tired	−0.195	0.544	−0.020	0.305	−0.175	−0.979	+0.933
Thirst	−0.045	0.888	0.000	0.224	−0.045	−0.816	+0.659
Nausea	−0.077	0.813	−0.050	0.370	−0.027	−0.904	+0.806
Clumsiness	−0.209	0.514	−0.054	0.275	−0.155	−0.910	+0.703
Reduced appetite	0.105	0.745	−0.048	0.404	0.057	−0.956	+0.939
Headache	−0.260	0.414	−0.022	0.241	−0.238	−0.882	+0.611
Weakness	−0.002	0.995	−0.128	0.473	0.126	−0.963	+0.932
Dizziness	−0.005	0.988	−0.052	0.403	0.047	−0.952	+0.933
Apathy	0.060	0.853	−0.019	0.373	0.041	−0.972	+0.965
Stomach pain	−0.117	0.718	−0.103	0.389	−0.014	−0.978	+0.765
Sensitivity to light	−0.138	0.669	−0.031	0.292	−0.107	−0.845	+0.674
Sensitivity to sound	−0.157	0.627	−0.047	0.298	−0.110	−0.851	+0.699
Shaking, shivering	−0.370	0.236	−0.076	0.290	−0.294	−0.958	+0.492
Confusion	−0.113	0.726	−0.071	0.390	−0.042	−0.961	+0.982
Depression	−0.160	0.618	−0.085	0.425	−0.075	−0.960	+0.984
Regret	−0.150	0.641	−0.061	0.431	−0.089	−0.980	+0.987
Heart pounding	−0.298	0.347	−0.067	0.384	−0.231	−0.995	+0.952
Sweating	−0.136	0.674	−0.061	0.479	−0.075	−0.964	+0.972
Vomiting *	−0.609	0.035	−0.032	0.179	−0.577	−0.944	−0.059
Heart racing	−0.211	0.511	−0.076	0.411	−0.135	−0.976	+0.962
Anxiety	−0.214	0.505	−0.065	0.466	−0.149	−0.990	+0.993

Reported r_P_ and *p*-value are from the original partial correlation, controlling for eBAC. Bootstrapping was conducted with B = 10.000 samples. A 99.8% CI_B_ (corresponding to *p* < 0.002) was used to correct for multiple comparisons. The r_PB_’s are significant if the BCa 99.8% CI_B_ does not contain zero, indicated by *. Abbreviations: r_P_ = partial correlation, eBAC = estimated blood alcohol concentration, SE = standard error, BCa = bias-corrected and accelerated, CI_B_ = bootstrapped confidence interval.
